# Supplementary material for: Association between oral microbiome and seven types of cancers in East Asian population: a two-sample Mendelian randomization analysis
Source: Front Mol Biosci. 2023 Nov 21;10:1327893. doi: 10.3389/fmolb.2023.1327893 (PMC10702768; doi:10.3389/fmolb.2023.1327893)
Supplement: Supplementary file 1 [file DataSheet1.ZIP › Supplementary Materials/MR plots of saliva/saliva═╝/Prostate cancer/pheno.1234_to_prostate cancer_forest.pdf]

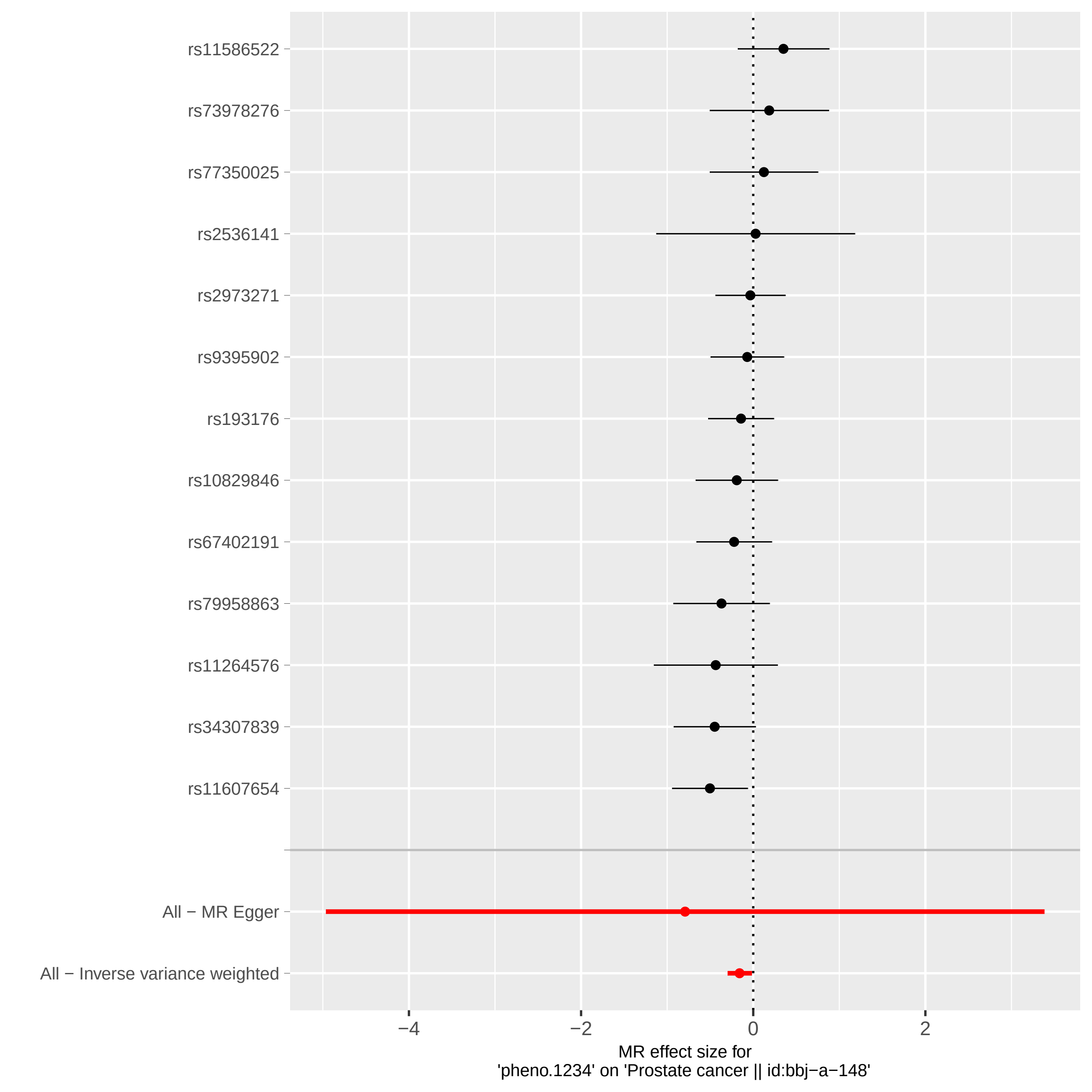

rs11586522
rs73978276
rs77350025
rs2536141
rs2973271
rs9395902
rs193176
rs10829846
rs67402191
rs79958863
rs11264576
rs34307839
rs11607654
All − MR Egger
All − Inverse variance weighted
−4
−2
0
2
MR effect size for
'pheno.1234' on 'Prostate cancer || id:bbj−a−148'
